# The Safe Assessment Form to Evaluate Risks (‘SAFER’) chart – a clinical practice evaluation study following introduction of electronic risk identification in pregnancies in Scotland

**DOI:** 10.3310/nihropenres.13791.1

**Published:** 2025-04-23

**Authors:** Alex Viner, Oscar Deeks, Pamela Nayyar, Jennifer Allison, Sarah Murray, Katherine Ainslie, Neil Cockburn, Richard Lilford, Brian Magowan

**Affiliations:** 1 Queen Elizabeth University Hospital, Glasgow, Scotland, UK; 2 School of Health Sciences, University of Birmingham, Birmingham, England, UK; 3 Victoria Hospital, Kirkcaldy, Scotland, UK; 4 Centre for Reproductive Health, The University of Edinburgh, Edinburgh, Scotland, UK; 5 NHS Borders, Melrose, Scotland, UK

**Keywords:** Electronic screening, paper screening, electronic health records, maternity guideline implementation

## Abstract

**Background:**

It is easy to overlook risk factors that require specific healthcare actions. This is particularly true in maternity care, which deals with a natural process where risk might be distinguished from normality at many points in the care pathway. In this paper, we describe the effects of clinical decision support, first in the form of paper checklists and then in the form of an electronic checklist to screen for risks of (1) venous thromboembolism (VTE), (2) intrauterine growth restriction (IUGR), (3) high body mass index (BMI), and (4) gestational diabetes mellitus (GDM). Here, we track the effects of different screening algorithms introduced on different media (paper versus computer).

**Methods:**

We screened sequential maternity records at three time points: baseline, following the introduction of a paper checklist, and following the introduction of the electronic system. First, we examined (at each time-point) the proportion of pregnancies appropriately screened at each time point. Second, we examined the proportion of correct actions taken following a positive screening result. The study was conducted at a District General Hospital in Scotland between 2011 and 2015, which covered the introduction of the above system to screen patients and suggest appropriate management for positive cases.

**Results:**

We found that the introduction of a paper checklist was associated with an increased proportion of pregnancies appropriately screened and correct actions taken contingent on positive screening. These trends continued after the introduction of the electronic prompts.

**Conclusions:**

Compliance with maternity guideline recommendations for VTE, high BMI, high risk of fetal growth restriction, and GDM improved over time with the introduction of paper and electronic prompts.

**Tweetable abstract:**

Electronic maternity screening is significantly superior to no screening or paper-based screening for VTE, BMI and GDM.

**Data Sharing Statement:**

Nil additional unpublished data from the study are available.

## Plain language summary

Maternity care identifies risks in an essentially normal population. However, it is easy to overlook the risk factors among the masses of information collated. To mitigate this problem, we implemented a checklist system. These checklists were first implemented in paper form and then in electronic form. These checklists covered (1) the risk of venous thrombosis and embolism, risk of stillbirth, and risks related to body mass index and gestational diabetes.

We sampled three sets of case notes at the end of calendar years 2011, 2014, and 2015, corresponding to points prior to paper checklists, after implementation of paper checklists, and after implementation of the electronic system. Each set of case notes was examined to determine the rates of adherence to the established standards of care related to the above risks.

## Introduction

Clinical decision support has a long history. MacDonald published a paper in the New England Journal of Medicine on decision support to prompt clinicians as long ago as 1976 ^1^. His paper addressed what he called the “non-perfectibility” of humans. Since then, numerous methods have been developed to provide clinical support. Computerised decision support for medical prescribing is an archetypal and well-studied example of such decision support ^2^. We shall refer to these decision support systems that identify a risk and then suggest an action to mitigate the risk as ‘prompts’.

Despite their widespread use in medicine and prescribing, systems to prompt clinicians to take action have not been widely used in maternity care. This is in contrast to systems to inform and support decision making once a risk has been identified, for example, to help people choose between vaginal and caesarean birth plans or whether or not to undergo invasive prenatal testing for congenital conditions ^3^. A randomized trial in 1992 showed that use of structured data and prompts could improve adherence to established principles of safe/effective antenatal care ^4^. Only seven studies, all in Low or Middle-income countries, have sought to replicate these findings over the past thirty-one years (see discussion).

In this study, we evaluated a program to implement clinical prompts in a District General Hospital (Borders General Hospital) in Scotland. Clinicians were prompted to act where necessary, in accordance with four sets of evidence-based national guidelines. The prompts were designed to (1) collect appropriate data, (2) alert clinicians to any risk, and (3) suggest contingent action when the risk is present. The study period was 2011 to 2015. During this period, changes were made in the “message and the medium” – different messages/prompts (e.g. for venous thrombosis and diabetes) were introduced at different times on different media (paper or computer). Initially, the prompts were paper-based in the form of a paper checklist that the clinician was required to complete for all the patients. At a later stage, the paper checklist was supplemented by an electronic form called the Safe Assessment Form (SAFER checklist) to Evaluate Risks. This phasing of prompts enabled us to track compliance with standards of care according to the particular standard and type of medium used to present the prompt to the clinician. More specifically, we tracked the extent to which clinicians complied with criteria for safe care over three time periods according to whether or not the particular prompt had been implemented and according to the method of presentation. After completion, the paper checklist or electronic print-outs were incorporated into clinical notes.

### Patient and Public Involvement

There was no Patient and Public involvement in this research study. This paper describes a retrospective, cross-sectional audit of a set of 300 case notes.

## Methods

### Overall design

We sampled three sets of case notes at the end of the calendar years 2011, 2014, and 2015, corresponding to points prior to paper checklists, after implementation of the paper checklists, and after implementation of the electronic (SAFER) system. Each set of case-notes was examined to determine the rates of adherence to established standards of care – thus we carried out three cross-sectional sets of observations or ‘audits.’

### The interventions

***Standards***

We established four sets of standards, each addressing one of the four clinical areas described below. Each standard contained two components.

(1) Risk assessment component and (2) contingent action component. The risk-assessment should be carried out on all patients and the ‘contingent action’ should be carried out in those cases where it is indicted on the basis of the risk-assessment – in other words contingent on the case being high risk. Risk assessment was based on a checklist of questions and observation of body mass index (BMI). The system algorithm then issues prompts based on certain data, either singly or in combination. A corollary of this sequence (assessment and action suggestion) is that, while all cases sampled would be eligible for the assessment/screening step, only a subset (those who screen positive) would be eligible for the contingent action step, a point to which we shall return.

The four standards, all based on national guidelines, were:-

1) Venous thromboembolism (VTE), based on the Royal College of Obstetricians and Gynaecologists (RCOG) guidelines ^5^. VTE is the leading cause of direct maternal death in the UK ^6^. There are three time points at which a VTE prompt is appropriate – antenatally (at the initial ‘booking’ visit or, when a patient is admitted) and postnatally. However, these were not introduced simultaneously, as described below.
2) Intrauterine growth restriction (IUGR). There are many factors that increase the risk of intrauterine growth restriction, which is a precursor to poor perinatal outcome, and contingent actions can be taken to reduce this risk ^7^. This standard is also underpinned by RCOG guidance ^8^. There are many possible actions that might be prompted when the risk of IUGR is detected, but here we concentrate on serial growth scans because of consensus on this point.
3) Body Mass Index (BMI). Again, this standard is covered by an RCOG guideline, itself based on the need to reduce obesity-associated outcomes including gestational diabetes, pre-eclampsia, perinatal death and miscarriage ^9^. There are three actions that follow positive screening for BMI-behavioural change support: performance of an oral glucose tolerance test (OGTT), and prescription of certain antibiotics.
4) Screening for gestational diabetes (GDM) – a topic related to obesity and again supported by guidance and evidence ^10, 11^ that adverse pregnancy can be avoided ^12, 13^. We identified the performance of a Glucose Tolerance Test as a definitive test as the appropriate action for women screening positive.

***Phasing of intervention***

The VTE prompt is invoked in three stages in the patient pathway (above). The postnatal VTE prompt was implemented many years prior to the initial observation at the end of 2011. Thus, there was no pre-implementation period for this component of the VTE prompt. Antenatal VTE prompts, however, were implemented soon after 2011 and before the second observation period in 2014. The IUGR prompt was implemented after 2011 (in 2013) and before 2014. However, the BMI and GDM prompts were only implemented in 2015.

**Figure 1.**
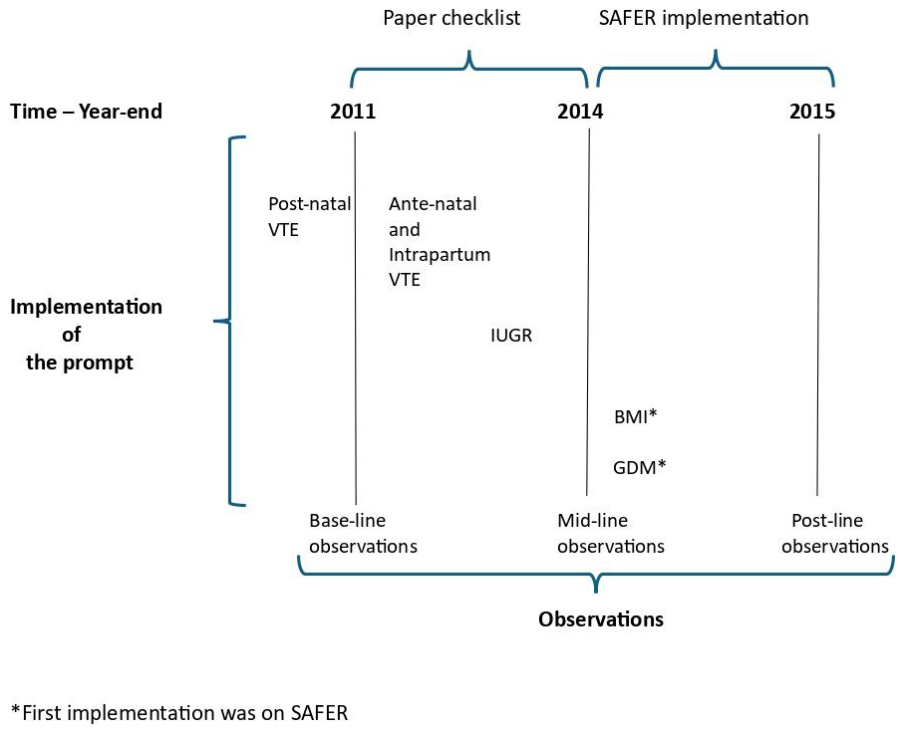
Time chart – interventions and observations.

The phasing of interventions (prompts) and observation periods (audits) are shown in Figure 1.

We also provide a summary of the prompt media used across each observation point in Table 1.

**Table 1.** Screening methods carried out at the selected time intervals.

	2011	2014	2015
**Antenatal VTE**	No formal screening	Paper checklist	SAFER
**Postnatal VTE**	Paper checklist	Paper checklist	SAFER
**IUGR**	NA *	Paper checklist	SAFER
**BMI**	No formal screening	No formal screening	SAFER
**Gestational Diabetes**	No formal screening	No formal screening	SAFER

*Guideline not introduced until 2013

***Data collection points and missing data***

The observations available for analysis were as follows:

1)   Postnatal VTE. Collected at baseline and midline but implemented before the 2011 baseline.

2)   Antenatal VTE. Collected at baseline, midline and endline.

3)   IUGR. Not collected at baseline (the intervention was not implemented until 2013 and, unfortunately, data were missing for the effect of moving from no prompts to paper prompts. Collected at midline and endline.

4)   BMI and GDM. Collected at baseline, midline and endline.

***Observations***

**Figure 2.**
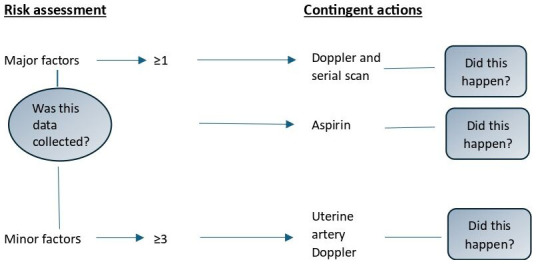
Representation of the small-for-gestational-age guideline.

Audits were carried out in the form of cross-sectional observations at the above three time points. At each observation time point, we sampled 100 consecutive case notes from the singleton pregnancies. Completed checklists or electronic printouts were included in these case notes. These documents and corresponding case notes were scrutinized to observe whether the required data had been collected and whether any contingent actions prompted by the system had been carried out. For example, in a set of 100 case notes, we first observed whether the risk factors for fetal risk were collected. Then, if a prompt was issued on the basis of this information, we recorded if that contingent action (in this scenario, case-serial scans) was taken. The denominator for this contingent action would be the number of cases where an action was required according to “opportunity for error” principles ^14^. We represent this dual quality assessment process in Figure 2 in relation to the IUGR risk factor. The sample size of 100 in each group provides 90% power to identify a 20% difference in compliance with a screening standard across any two time periods. Precision was lower with respect to contingent actions.

### Analysis

To investigate the relationship between the different media used for screening algorithms in maternity care and the prompt completion proportion, we used logistic regression modelling. We used descriptive statistics to summarize the proportions of the prompts completed successfully for each clinical area, split into each time point, where the denominators are the total number of participants at each time point. We summarized two different proportions for each clinical area: the proportion of correctly completed risk assessments, and the proportion of contingent actions completed successfully. All analyses were performed using R 4.4.1.

Two logistic mixed-effects regression models were used to estimate the association between the medium of the prompt and how successfully it was completed. One model was used to examine the proportion of successfully completed risk assessments and the other to examine the proportion of successfully completed contingent actions. We pooled the clinical areas to estimate the mean difference in the proportion of risk assessments and contingent actions completed successfully across patients and clinical areas. The dependent variable was coded as 1 if the relevant part of the prompt was completed successfully and 0 if it was not. This model aims to find a more succinct estimate of whether there is an association between the medium of the prompt and the chance of the prompt being completed successfully. We included the following explanatory variables: type of prompt (the explanatory variable of interest), clinical area (VTE, IUGR, BMI, GDM), and year (2011, 2014, 2015). There is collinearity between the year 2015 and the medium type of a SAFER prompt, as in 2015, SAFER prompts were implemented across all clinical areas. We also included a mixed-effect random variable in one of our models to account for the possibility that each person could have multiple records within each clinical area related to different contingent actions. Using the values in Table 2, we adjusted for the average age and BMI each year. We also tested the collinearity of the variables included in the models ( Table 3), where the generalized VIF values were < 3, showing low to moderate collinearity.

**Table 2.** Demographics – The Age and BMI of the three cohorts.

Cohorts	2011	2014	2015
**Number of patients (n)**	100	100	100
**Mean Age in years (SD)**	28.4 (6.3)	27.2 (6.3)	28.3 (5.5)
**Mean BMI (SD)**	26.6 (6.1)	25.6 (6.5)	27.3 (7.2)

**Table 3.** GVIF value.

Risk Assessment model	GVIF	GVIF ^(1/2( *Df)) †^
**Prompt type**	2.86	1.30
**Year**	1.80	1.34
**Area**	1.68	1.09

^†^ Df = degrees of freedom * Generalised Variance Inflation Factor

## Results

Among the 300 participants in the study, the mean age was 28.0, and the mean BMI was found to be 26.5. These values were similar across the three study epochs ( Table 2).

### Descriptive statistics results

As stated, to standardize, we simplified the questions to the same two questions for each clinical area: “Was the correct risk assessment made?” and “Was the correct contingent action applied when appropriate?”. The question related to risk assessment contained a value for all 100 participants over each of the three time points. For this value to be positive for each participant, the risk assessment must have been correct. If either of these criteria was not met, it was recorded as not having been completed when calculating the proportion. The question relating to contingent actions only contained values where contingent actions were needed, with the proportion being the total number of contingent actions that were completed correctly for each clinical area out of the total number of actions that should have been completed. Note that there are some areas where participants can have multiple treatments, causing the total number of contingent actions to be larger than the number of eligible people. The results for all the four conditions are presented in Table 4.

**Table 4.** Proportions of correctly completed risk assessments and contingent actions across each time point.

Antenatal and intrapartum VTE	2011 (non-formal)	2014 (paper checklist)	2015 (SAFER)
Did we make the correct risk assessment?	0/200 (0.000)	69/200 (0.345)	192/200 (0.965)
Was the correct contingent action applied when appropriate?	0/1 (0.000)	17/24 (0.708)	20/33 (0.667)
Postnatal VTE	2011 (paper checklist)	2014 (paper checklist)	2015 (SAFER)
Did we make the correct risk assessment?	93/100 (0.930)	97/100 (0.970)	98/100 (0.980)
Was the correct contingent action applied when appropriate?	60/62 (0.968)	48/48 (1.000)	55/57 (0.965)
IUGR	2011	2014 (paper checklist)	2015 (SAFER)
Did we make the correct risk assessment?	NA	93/100 (0.930)	97/100 (0.970)
Was the correct contingent action applied when appropriate?	NA	70/81 (0.864)	81/87 (0.931)
BMI	2011 (non-formal)	2014 (non-formal)	2015 (SAFER)
Did we make the correct risk assessment?	82/100 (0.820)	97/100 (0.970)	97/100 (0.970)
Was the correct contingent action applied when appropriate?	27/86 (0.314)	62/73 (0.849)	110/127 (0.866)
GDM	2011 (non-formal)	2014 (non-formal)	2015 (SAFER)
Did we make the correct risk assessment?	98/100 (0.980)	100/100 (1.000)	99/100 (0.990)
Was the correct contingent action applied when appropriate?	4/34 (0.118)	26/31 (0.839)	39/52 (0.750)

***Antenatal VTE prophylaxis***

There was a significant improvement from 2014 to 2015 when there was a change from using a paper checklist to an electronic SAFER prompt. In 2014 34.5% of the risk assessments were completed correctly, compared to 96.5% of SAFER prompts completed correctly in 2015. However, there appeared to be little difference in the proportion of correct contingent actions applied when the SAFER prompt was introduced.

***Postnatal VTE prophylaxis***

However, the implementation of *postnatal* VTE preceded the first observation period ( Figure 1). There appears to be a ceiling effect with how often postnatal VTE risk assessments are completed correctly, as 93%, 97%, and 98% of the risk assessments were completed correctly in 2011, 2014, and 2015, respectively. The same is the case for treatment of postnatal VTE, where 96.8%, 100%, and 96.5% of the indicated treatments were completed successfully. This contrasts with the pattern for *antenatal* VTE above, where high compliance was delayed until after its guideline came into operation.

***IUGR***

There were no guidelines in 2011 regarding IUGR risk assessments nor were data collected at this time point. In 2014 and 2015, 93% and 97% of IUGR risk assessments, respectively, were completed correctly. There is some suggestion of an association between improvement with respect to this prompt type when considering whether the correct contingent actions were applied, where 70/81 (86.4%) contingent actions were applied successfully in 2014 and 81/87 (93.1%) in 2015 when the SAFER prompt was implemented.

***BMI***

When observing both the proportion of BMI risk assessments completed correctly and the proportion of contingent actions applied correctly, there appears to be an increase from 2011 to 2014, although there are no formal systems (checklists or SAFER electronic systems) used in either year. Contingent action, for example, increased from 31% to 85%. This may suggest that there is a temporal association, net of any intervention, towards improved surveillance over those years. There appears to be no significant difference between 2014 and 2015 once SAFER prompts had been implemented, with differences of 0 and 2 percentage points for assessment and contingent action, respectively.

***GDM***

Similar to BMI, no checklist intervention was implemented before the SAFER intervention between 2014 and 2015. Again, the proportion of GDM risk assessments that were completed correctly was very high in all of our samples. Although the proportion of correctly completed risk assessments is lower post-SAFER implementation in comparison to 2014 before prompts were implemented (100% (2014) and 99% (2015), the difference in these proportions is too small to support any conclusions. This is another example of the ceiling effect that appears when looking at how often risk assessments are correctly completed. We also observed no improvement with respect to the proportion of contingent actions completed correctly when comparing 2014 to 2015; however, there was a large increase from 2011 to 2014, reinforcing the suggestion that there may be an association between the time in which the risk assessment was used and an improvement in completing the risk assessments successfully, that is, a temporal trend.

### Logistic regression results

***Risk assessment model***

Using the coefficients from Table 5, we can calculate the difference in the absolute probabilities of risk assessments being completed correctly between different media of prompt. We begin by examining the differences between non-formal screening methods and paper checklists. There was an absolute difference in probabilities of 0.078 (7.8%) between risk assessments being completed correctly using non-formal versus paper prompts.

**Table 5.** Risk assessment model coefficients and CI’s.

	Coefficient Estimate	Odds ratios	95% Confidence intervals
Intercept	2.47	11.81	(7.03,19.86)
Medium_paper	5.14	171.53	(58.67,501.55)
Medium_SAFER	6.83	922.27	(304.01,2797.85)
Year_2014	-0.82	0.44	(0.28,0.69)
Area_Diabetes	2.21	9.10	(2.69,30.82)
Area_IUGR	-4.50	0.01	(0.00,0.04)
Area_VTE	-6.36	0.00	(0.00,0.00)

After this, we looked at the difference between the proportion of completed paper checklists and SAFER prompts, of which there was an absolute difference of 0.006 (<1%). This value is too small to be significant given our sample size, and is affected by the ceiling effect stated earlier.

The use of SAFER prompts versus non-formal screening techniques was associated with a higher proportion of correctly completed risk assessments. There is an absolute difference in probabilities of 0.08 (8%) between risk assessments being completed correctly using either non-formal prompts or SAFER prompts.

***Contingent action model***

The first comparison of contingent actions was made between non-formal screening techniques and paper checklists using the values from Table 6. There was an absolute difference in probabilities of 0.05 (5%) between the probability that a contingent action was completed correctly using paper-checklists versus using non-formal screening techniques.

**Table 6.** Contingent action model coefficients and CI’s.

	Coefficient Estimate	Odds ratios	95% Confidence intervals
Intercept	-3.37	0.03	(0.02,0.05)
Medium_paper	1.77	5.87	(3.96,8.69)
Medium_SAFER	1.56	4.78	(3.16,7.23)
Year_2014	0.09	1.09	(0.71,1.68)
Area_Diabetes	1.28	3.58	(2.54,5.06)
Area_IUGR	1.07	2.92	(2.11,4.03)
Area_VTE	0.44	1.55	(1.16,2.07)

When comparing the proportion of successfully completed contingent actions between the prompt medium of paper and SAFER prompts, we obtained a log odds estimate of 0.2058, which corresponds to a difference in probabilities of -0.007 (-<1%) between paper and SAFER prompts. This interpretation suggests that there is a slightly smaller chance that a SAFER prompt will have a successfully completed contingent action; however, this value is too small to be significant. As shown in the results, there is a very large increase in the absolute difference between informal screening techniques and the other two prompt media.

After adjusting for confounders in our model, the use of SAFER prompts over non-formal screening techniques was associated with a higher proportion of successfully completed contingent actions. There is an absolute difference in probabilities of 0.10 (10%) when comparing the probability of a contingent action being completed successfully between non-formal screening techniques and SAFER prompts.

One limitation of these models is that because all SAFER prompts were implemented in 2015, we were unable to account for the confounding effect of time; therefore, the odds ratios and absolute differences in probability include any confounding effect of time from 2011 to 2015. Even with this in mind, we are still seeing an increase in the proportion of prompts being completed correctly, which is a positive sign that risk screening in maternity care is improving, even if we cannot isolate the cause of this specifically.

## Discussion

### Trends in the data

A clear trend of improvement is seen across epochs: the proportions screened and proportions where the correct action was taken increased progressively from one epoch to the other. Only in the case of postnatal VTE is there no trend; this results from high baseline performance, leaving almost no headroom for further improvement. A checklist for postnatal VTE assessment preceded that of 2011; hence, our first set of observations. Thereafter, an antenatal VTE assessment was implemented, with subsequent improvement in assessment and contingent action. This pattern is certainly consistent with an intervention effect, but other explanations are plausible. One such explanation is the so-called rising tide phenomenon ^15^ where concern across a social system prompts general improvement *and* specific interventions. The headroom for improvement from the latter is reduced because of spontaneous improvements across the system. 

### Limitations and causal inference

There is little doubt that real improvement occurred over time; precision is low with respect to contingent actions, but the pattern in the data is highly consistent. The question is whether these changes were causal or resulted from other factors that created a secular trend. The fact that changes in the medium and message did not correspond to the dataset provides some clues. The improvement in adherence with respect to GDM and BMI over both earlier epochs, despite not using paper checklists, provides some evidence for a secular trend. On the other hand, as stated, the VTE data point towards a causal explanation since antenatal and intrapartum performance improved only after the checklists were introduced. The rapidity of change - over only one year - for standards where ceiling effects were not in evidence may provide at least some evidence that the effects were (at least partially) causal. However, the data presented here have limitations in the sense that no data were collected for IUGR in 2011. The 1992 randomized trial of prompts quoted in the introduction found that both paper and computer prompts caused a sharp increase in compliance with quality standards compared to no prompt at all. The results regarding antenatal VTE were consistent with these earlier findings.

### Relevance

While our study has significant limitations, it is one of only a few studies evaluating prompts in antenatal care. A recent systematic review summarised the world’s literature on decision support in maternity care ^16^. This literature described evaluation of many different types of decision support, covering decision aids for patient choice, prescribing systems, risk scores and decision prompts (covered here). The decision prompts covered postnatal and intrapartum care as well as single and multiple issues. We found seven studies (all from low- and middle-income countries) that evaluated multiple antenatal prompts, analogous to the study cited here ( Table 7). The findings, taken in the round, confirm our findings that antenatal decision support in the form of prompts is associated with improved compliance with criteria of effective antenatal care. The prompt systems supplemented pre-existing paper records. A much neater solution is to integrate prompts into an electronic record system. Since our study, electronic maternity records have been widely adopted in the UK. Thus, there is a need to evaluate the performance over the years following our observations, during which electronic records, some of which incorporate prompts, have been introduced. To gain an insight into the causal relationship between the improvement of completion and correctness of risk assessments and the contingent actions that follow, there should be a more in-depth analysis of a larger dataset that is able to account for all the confounding factors.

**Table 7.** Studies evaluating multiple antenatal prompts.

Authors	Intervention	Study Type	Location	Findings
Lilford, *et al.* 1992 ^4^	Unstructured notes, structured notes and computer system	Individual patient 3-way RCT	England	Structured histories and antenatal prompts improved adherence to care processes.
Horner, *et al.* 2013 ^19^	eHealth decision support system – compliance to care protocols	Before-and-after	South Africa	eHealth improved compliance with standards over a checklist system.
Venkateswaran, *et al.* 2022 ^20^	Digital clinical support vs. paper records	Cluster RCT	Palestine	Decision support superior for most process outcomes.
McNabb, *et al.* 2015 ^21^	Use of an app with decision support	Before-and-after	Nigeria	Most process outcomes improved.
Maïga, *et al.* 2023 ^22^	Digital clinical support	Cross-sectional, post-intervention, observational study	Burkina Faso	No difference in quality-of-care scores, but counselling and malaria prophylaxis improved in intervention clinics.
Benski, *et al.* 2017 ^23^	Mobile health system	Cross-sectional pilot study	Madagascar	The mHealth system studied is a feasible method for collecting large amounts of patient information.
Saronga, *et al.* 2017 ^24^	Electronic clinical decision support system	Quantitative pre- and post- intervention study	Tanzania	Marginal improvement in individual process quality variables (e.g. history taking). No significant improvement on overall process quality of care.
Ahmad, *et al.* 2016 ^25^	Educating on correct procedure for documenting VTE assessments	Before-and-after	England	Increase in completion of VTE assessments on EPR for the duration of the study.

The introduction of maternity records is a significant implementation that has taken place in the absence of any evaluation of which we are aware. Therefore, it is critical to evaluate these new systems. While the data presented here suggest that incorporating the above prompts in new electronic maternity systems is a good idea, there is no certainty that they were optimally configured for maximal impact. Electronic notes provide the basis for examining different formats and a wider set of prompts than we have examined, including blood test results (e.g., rhesus disease/anemia), medical disorders, medicine prescriptions, and mental health problems. There is a real risk of information overload with modern electronic systems ^17, 18^ and it will be important to study the effect of further formal systems and design systems to suit human users, not the other way around.

## Conclusion

Our retrospective study provides evidence on the value of prompts, particularly electronic prompts. To date, our study represents the only formal evidence relating to prompts incorporated in modern maternity electronic notes.

## Ethics and consent

The need for ethical approval and consent were not required. The local audit committee, NHS Borders Clinical Governance and Quality ruled on 13/01/2017 (application reference number 2016-743) that no formal ethics approval was required. There were no participants involved as the study was an audit to evaluate service improvement. We comply with guidance in paragraph 79 of the General Medical Council’s Confidentiality: good practice in handling patient information ( https://www.gmc-uk.org/professional-standards/the-professional-standards/confidentiality/ethical-and-legal-duties-of-confidentiality) which states that “Anonymised information will usually be sufficient for purposes other than the direct care of the patient and you must use it in preference to identifiable information wherever possible.” The information we use is totally anonymised. We also comply with paragraph 81 of the guidance, “The Information Commissioner’s Office anonymisation code of practice (ICO code) considers data to be anonymised if it does not itself identify any individual, and if it is unlikely to allow any individual to be identified through its combination with other data.” The data contains no information that would allow it to be linked back to patient notes. Furthermore, we also comply with paragraph 14g of the General Medical Council’s Confidentiality: good practice in handling patient information ( https://www.gmc-uk.org/professional-standards/the-professional-standards/confidentiality/ethical-and-legal-duties-of-confidentiality).

## Data Availability

Fighshare: SAFER study_audit case notes, DOI: https://doi.org/10.6084/m9.figshare.28483838.v2 ^26^. The project contains the following underlying data: SAFER study_audit case notes.xlsx Data are available under the terms of the Creative Commons Attribution 4.0 International license (CC-BY 4.0).
