# Supplementary material for: The Safe Assessment Form to Evaluate Risks (‘SAFER’) chart – a clinical practice evaluation study following introduction of electronic risk identification in pregnancies in Scotland
Source: NIHR Open Res. 2025 Jul 16;5:37. Originally published 2025 Apr 23. [Version 2] doi: 10.3310/nihropenres.13791.2 (PMC12531618; doi:10.3310/nihropenres.13791.2)
Supplement: Peer review history

# Reviewer report: Reviewer response for version 2

**DOI:** 10.3310/nihropenres.15256.r37070

**Published:** 2025-10-16

**Authors:** Abi Merriel

**Affiliations:** 1 University of Liverpool, Liverpool, UK

Thank you for the opportunity to review this study, about comparing three timepoints investigating no, paper and computer based decision support for core obstetric risk assessments. I think that the paper is well written and yields findings which are particularly useful in the current climate. With the need to embrace and use electronic health records to enhance care and ensure that we systematically identify and minimise risk. 

 My main query was how you decided to review 100 cases per epoch. It feels as though this was decided out of convenience? 

 It seems that this was a quality improvement project, which was conducted many years ago. Whilst i agree the findings still relevant it would be useful to have a bit more of the context of why this project was carried out.

 I have just a few minor points. 

 1) page 5 in the section where you are discussing standards. I am a little confused by the BMI section. I'm not clear that antibiotics are routinely prescribed for BMI. I think it could also do with the actions for BMI being better punctuated to help with flow. 

 2) for me i felt that the descriptive statistics part would sit better in the analysis section rather than the results - It felt that i was going too and fro. 

 3) I would have appreciated an explanation of the VIF in the methods, so that I was expecting to see it as a core way for results to be presented. 

 Overall, I enjoyed reading the paper and it has useful messages - especially the final message to build these risk assessment tools into the electronic maternity notes.

Is the work clearly and accurately presented and does it cite the current literature?

Yes

If applicable, is the statistical analysis and its interpretation appropriate?

Yes

Are all the source data underlying the results available to ensure full reproducibility?

Yes

Is the study design appropriate and is the work technically sound?

Yes

Are the conclusions drawn adequately supported by the results?

Yes

Are sufficient details of methods and analysis provided to allow replication by others?

Yes

Reviewer Expertise:

maternity research

I confirm that I have read this submission and believe that I have an appropriate level of expertise to confirm that it is of an acceptable scientific standard.

# Reviewer report: Reviewer response for version 2

**DOI:** 10.3310/nihropenres.15256.r36973

**Published:** 2025-09-23

**Authors:** Dimitri Abrahamsson

**Affiliations:** 1 University of California San Francisco Department of Obstetrics Gynecology and Reproductive Sciences (Ringgold ID: 271614), San Francisco, California, USA

The authors present an interesting retrospective observational study that aims to evaluate the implementation of clinical decision support prompts for various pregnancy-related risk factors across three time periods (2011, 2014, 2015). The study addresses a critical gap in maternity care regarding clinical decision support systems with potential implications for patient health. The findings of the study are interesting, and I believe that they will be of interest to other researchers in the field. That being said, I think some areas in the manuscript would benefit from additional clarifications.

 Major comments:

 Causal inference

 Although acknowledged by the authors, the causal link is somewhat weak. The retrospective, cross-sectional design of the study prevents the authors from establishing causality. This appears to be an inherent deficiency of the study and I am not sure how it can be addressed within the current framework. A more robust approach would perhaps involve continuous data collection before and after each intervention.

 Sampling and abstraction

 The methods would benefit from some additional details. It is unclear how the 300 cases were sampled. Were they selected randomly? I am a little confused whether this was done randomly or consecutively.

 Missing demographics

 Table 2 is rather frugal regarding demographics. The table would benefit from additional variables such as parity, gravidity, level of education, and household income.

 Sample size and statistical power

 The sample size of 100 cases per time point is moderate and could potentially limit statistical power. The manuscript would benefit from a power calculation to determine the effect sizes that can be observed with this cohort size and composition.

 Interpretation of findings

 The authors should consider expanding the discussion section to include potential barriers to implementing contingent actions and suggestions for addressing them.

 Minor comments:

 Ethics statement

 While ethics approval was considered unnecessary, it would be helpful to know how data privacy and confidentiality were ensured.

 Terminology

 Perhaps it would be helpful to define “contingent actions” early on in the manuscript so that it’s easier for the reader to follow along.

Is the work clearly and accurately presented and does it cite the current literature?

Yes

If applicable, is the statistical analysis and its interpretation appropriate?

Partly

Are all the source data underlying the results available to ensure full reproducibility?

Partly

Is the study design appropriate and is the work technically sound?

Partly

Are the conclusions drawn adequately supported by the results?

Partly

Are sufficient details of methods and analysis provided to allow replication by others?

Partly

Reviewer Expertise:

My area of expertise is in environmental exposures and their effects of perinatal outcomes such as preterm birth and gestational diabetes.

I confirm that I have read this submission and believe that I have an appropriate level of expertise to confirm that it is of an acceptable scientific standard, however I have significant reservations, as outlined above.

# Reviewer report: Reviewer response for version 1

**DOI:** 10.3310/nihropenres.14980.r35641

**Published:** 2025-06-09

**Authors:** Mxolisi W Ngwenya

**Affiliations:** 1 University of Limpopo, Mankweng, Limpopo, South Africa

Thank you for the opportunity to review the paper entitled, *“ **The Safe Assessment Form to Evaluate Risks (‘SAFER’) chart – a clinical practice evaluation study following introduction of electronic risk identification in pregnancies in Scotland” *** The present a novel approach towards early detection of high-risk conditions in pregnancy. The study findings are compelling and are of significance in advancing maternal health in clinical practice. However, I have a few comments and suggestions for the authors.

**Abstract**

- The abstract is well-designed and highlight some of the significant information to the readers. However, to enhance its readability I suggest that in the background should highlight the briefly indicate a research gap to why the study was conducted, particularly in Scotland. In methods, I suggest the authors highlight the research methodology followed (If it is quasi-experimental, please indicate).
- Drawing from the abstract conclusion, the authors indicated that the use of the risk assessment is associated with positive outcomes. I suggest they also indicate the implications of this tool (digital/paper-based) in clinical practice and policies.

**Introduction and background**

- The introduction and background, mostly focuses on describing the current study process and implementation of the SAFER tool. I suggest the authors provide more detailed information of the importance of the clinical decision tools particularly in maternity units. Moreover, the authors could further describe in detail the potential benefits of clinical decision support tools on pregnancy outcomes based on the existing literature.
- In addition, since the tool was then supplemented to be digital thereafter. I strongly suggest the authors add literature to why they opted to supplement it into an electronic form. For instance, the authors include the benefits and positive implications of digital health in the current era.

**Methods**

- I suggest that the authors should add the research design followed to enhance the study credibility and reproducibility.
- I suggest the authors also indicate on how they sampled the 300 cases, which sampling technique did they follow. This would the credibility of the study and enhance its understandability to the readers.
- The authors indicated that they delved into four standards namely, BMI, VTE, IUGR and DM. They devised a set of questions to screen those conditions. I suggest they indicate the process of how the set of questions were developed and validated (i.e. if experts were involved in the content and face validity of the devised questions). if the tool was adapted the authors could indicate so.
- In addition, although the study is already implemented. I am just wondering if the authors have a particular reason to why hypertensive disorders in pregnancy were excluded, since it also one of the leading causes of maternal mortality worldwide.
- I suggest the authors provide a detailed inclusion and exclusion criteria to which pregnant women were eligible to this intervention. This would enhance the study quality and methodological rigor.
- Kindly indicate the time frame if the files were from year-round or only a couple months in each year. (I.e. The data was collected from Jan-Dec in 2011, 2014 and 2015). This could also be well reflected in figure 1.
- In data collection, kindly indicate whether the screening was normally done on hospital visits by all pregnant women or on admission or if this tool was incorporated in a routine practice within the hospital. Kindly provide a detailed methods to enhance its methodological rigor.
- Kindly indicate on how the duplication of cases was addressed. For instance, if some of the patients were re-admitted.

**Results**

- Please include the demographic data of the participants throughout the years 2011, 2014 and 2015. This will also assist to identify the trends on the demographics associated with the high-risk conditions. (Age, parity, Gravidity, and educational level, etc) This could also reflect the effectiveness of the tool in identifying demographic factors predisposing those pregnant women at risk at developing the identified conditions.

**Discussion**

- One of the study objectives as highlighted by the authors was to ‘ *describe the effects of clinical decision support…*’ However, the discussion lacks implication of the use of this particular tool in clinical practice. I suggest the authors indicate to whether the use of this tool enhance patient outcomes and improved quality of care. This could be also linked to the high compliance rate with the SAFER in 2015. Thereafter the authors could add more policy and clinical practice implications of introducing this particular tool in the maternity units.

Conclusion

- Similar to the abstract, I suggest that the authors indicate the implications of this tool (digital/paper-based) in clinical practice and policies in relevance to their findings.

Is the work clearly and accurately presented and does it cite the current literature?

Partly

If applicable, is the statistical analysis and its interpretation appropriate?

Yes

Are all the source data underlying the results available to ensure full reproducibility?

Partly

Is the study design appropriate and is the work technically sound?

No

Are the conclusions drawn adequately supported by the results?

Partly

Are sufficient details of methods and analysis provided to allow replication by others?

Partly

Reviewer Expertise:

Maternal and digital health

I confirm that I have read this submission and believe that I have an appropriate level of expertise to confirm that it is of an acceptable scientific standard, however I have significant reservations, as outlined above.

# Response

**Published:** 2025-07-03

**Authors:** Richard Lilford

**Affiliations:** University of Birmingham, Birmingham, England, UK

We thank the reviewer for their thorough consideration of our paper.

Abstract

 We have highlighted the methodology in the abstract. To remain within the word limit, we have mentioned the research the gap and the relationship between taking action and outcome in the Introduction.

Introduction

- Further detail on decision tools and relation to outcomes added.
- Potential advantages of digital over paper checklists and prompts added.

Methods

- Design added.
- We explicate the design by adding that the consecutive sampling was sequential from on a pre-defined date.
- We add that the checklist was implemented ‘verbatim’ form the published checklist with no adaptation, to the contingent action recommended by the Boolean (if/then) algorithm (as laid out in Figure 2).
- We did not include hypertension because this would have required a more complex algorithm on which there is less consensus. We have not altered the text because hypertension is one of many potential triggers for a prompt.
- We have not produced exclusion criteria since there was only one already listed as our inclusion criteria.
- We had stated that we sampled at the end of each calendar year and now add “November and December”.
- We now state “booking clinic” – which also answers the next question.

Results

- Since the cases were consecutive, they represent the users of the service. Our focus was clinical behaviour in terms of adherence to checklist and guidelines for contingent action.

Discussion and Conclusion

 We now specify the relationship between observed change in process (clinical behaviour) and outcome.

# Reviewer report: Reviewer response for version 1

**DOI:** 10.3310/nihropenres.14980.r35401

**Published:** 2025-05-24

**Authors:** Steven Ondersma

**Affiliations:** 1 Charles Stewart Mott Department of Public Health, Michigan State University, Flint, Michigan, USA

Clinician-directed prompts for risk screening, followed by care recommendations for positive screens, are a well-known approach to facilitating adherence to clinical practice guidelines. This interesting manuscript uses a retrospective review to examine changes in screening rates and subsequent recommended action for four specific pregnancy-related risks following implementation of prompts. In a sample of 300 case records, the authors find clear increases in adherence across the three observation points (2011, 2014, and 2015) in 2 of the 4 areas, and high levels of adherence at all observed points for the others. The authors appropriately consider challenges to causal inference but also suggest that the findings support the use of prompts, particularly in electronic format. The authors are quite correct that such techniques are an important area of study, and they make creative use of historical medical records. The measurement of appropriate actions with screen-positive patients, and not just rates of screening, is especially valuable. Unfortunately, multiple factors negatively affect the rigor and ultimately the impact of this effort.

1. The authors reference the clinical decision support and prompt literatures and suggest that little research has been done regarding prompts in antenatal care in the past several decades, particularly in high-income countries. More relevant research may be available in studies of quality improvement, practice guideline adherence, and Electronic Health Record (EHR)-based prompts.    
2. The timing of abstracted cases does not correspond with the introduction of the prompts into regular clinical practice; case notes were sampled at the end of three separate calendar years sometime after the changes had been introduced. This lack of resolution complicates causal inference, particularly compared to alternative methods such as continuous observation of cases before and after the change was introduced. This is particularly true given references to changes in the message over time (these changes and their timing are not specified) and the likely evolution of EHR risk screening and best practice prompts over time.
3. More detail is needed regarding how much reviewer judgment was needed when abstracting case notes. For example, did the case notes have clear checkboxes that drove ratings, or did reviewers interpret actions based on written notes? And if the latter, did the investigators use a clear manual and determine inter-rater reliability?
4. Were records selected consecutively based on dates, or using some other criteria?
5. Similarly, how were the four risk factors selected? Were other prompts introduced during the study period that were not selected for study?
6. The manuscript notes that paper checklists were supplemented by the SAFER electronic checklist. Does this mean that some elements of the risk screening in question continued to be done on paper?
7. EHRs regularly require screening for a wide range of potential risks and provide clinical decision/best practice alerts following a positive screen. Can the authors comment on the extent to which the high rates of adherence observed might be explained by increased use of required fields and best practice alerts in EHRs used in Scotland during the study period? Or the extent to which evidence-based prompts are already entering widespread use via EHRs?
8. The abstract should include the sample size and statistics to support the key findings. Please also edit the tweetable abstract to be more measured.

Is the work clearly and accurately presented and does it cite the current literature?

Partly

If applicable, is the statistical analysis and its interpretation appropriate?

Partly

Are all the source data underlying the results available to ensure full reproducibility?

Yes

Is the study design appropriate and is the work technically sound?

Partly

Are the conclusions drawn adequately supported by the results?

Partly

Are sufficient details of methods and analysis provided to allow replication by others?

Partly

Reviewer Expertise:

Risk screening and technology-based screening and brief intervention for pregnancy-related risks, particularly substance use.

I confirm that I have read this submission and believe that I have an appropriate level of expertise to confirm that it is of an acceptable scientific standard, however I have significant reservations, as outlined above.

# Response

**Published:** 2025-07-03

**Authors:** Richard Lilford

**Affiliations:** University of Birmingham, Birmingham, England, UK

We thank the reviewer for their attentive reading of our paper and for insightful suggestions.

1. We may have missed other literature. Our search would have included the quality literature covering antenatal care. It was done independently of a recent literature review cited in our article (Cockburn et al. 2024) and our search included all articles on prompts/action suggestions cited in this paper.
2. Sampling at the end of calendar years versus continuous observation. Doing so would have introduced (additional) sources of bias. Had we done so, we would have needed an adjustment period for the clinicians to get used to each new prompt system as it was introduced. More observations would have been required.
3. Role of reviewer judgement. This relates to the " observations” subheading under methods. As stated, we retrieved the case notes. The case notes included the paper checklists that we introduced in the earlier epoch and the electronic print outs from the later epoch. We now make that clearer. We then go on to specify that the checklists and electronic printouts were scrutinised to determine whether the data was collected. Then the case notes themselves were examined to see whether the contingent actions were followed. We have rewritten this part to make this clearer. Thank you. We then add “Thus the data collected were based on clear boxes on the paper or electronic checklist, while the contingent actions were extracted from the case notes.”  Then in the ‘limitations’ section of the discussion we add “The elicitation of data collected by clinical staff was based on clear check boxes. However, the contingent actions had to be ascertained from case notes. While this exercise is not complex, we cannot exclude the possibility that some of the actions were overlooked (leading to underestimation of effects) and we did not have resources to duplicate observations. However, the finding of ceiling effects for some observations suggests that few, if any, of the contingent actions were missed”. Thank you for this observation.
4. Case notes selection. Again, thank you. We have clarified that they were consecutive.
5. Selection of prompts. We now add “The clinical service decided to implement four sets of standards. They were selected because they all had national endorsement (see below). We therefore examined these standards.”
6. Paper checklists were “ **replaced**” by electronic checklists. Thank you - we should have been clearer.
7. High rate of adherence explained by increased use of required fields: evidence- based prompts on EHRs. We had made it clear that the systems described here were forerunners of electronic notes - first paragraph of ‘relevance’ section in the discussion. We had also discussed a more general trend under ‘causal inference.’
8. We have added sample size to the abstract. We have rewritten the ‘Results’ section to include the statistical findings. We agree that the tweetable abstract was too positive and have modified it.
